# Patients’ experiences with participating in a team-based person-centred intervention for patients at risk of or diagnosed with COPD in general practice

**DOI:** 10.1186/s40814-023-01398-9

**Published:** 2023-09-25

**Authors:** Beate-Christin Hope Kolltveit, Marit Graue, Christine Råheim Borge, Bente Frisk

**Affiliations:** 1 Department of Health and Caring Sciences, Western Norway University of Applied Sciences, Bergen, Norway; 2 Vossevangen Medical Center, Voss, Norway; 3 Department of interdisciplinary health sciences, University of Oslo, Oslo, Norway; 4 Department of Research, Lovisenberg Diaconal Hospital, Oslo, Norway; 5 Department of Health and Functioning, Western Norway University of Applied Sciences, Bergen, Norway

**Keywords:** COPD, Guided self-determination, General practice, Primary care, Qualitative

## Abstract

**Background:**

Symptoms and complications of chronic obstructive pulmonary disease (COPD) can affect daily activities and quality of life, and patients with COPD require long-term follow-up by their general practitioner. Providing patients with or at risk of COPD practical skills and motivation to improve their self-management is important. On this background, an interdisciplinary follow-up program was designed based on the Guided Self-Determination counselling method to facilitate problem-solving and mutual decision-making between healthcare professionals and patients. The aim of the study was to explore patients and healthcare professionals` experiences with the Guided Self-Determination-program to investigate feasibility issues.

**Methods:**

A qualitative design was used to get insights in the experiences of receiving the Guided Self-Determination counselling program. In total, 13 patients with COPD (mean age 71.7 ± 7.7 years) 4 were current smokers, and 7 at risk of COPD (mean age 54.1 ± 9.9 years) all current smokers, received the Guided Self-Determination program. The researchers performed individual semi-structured telephone interviews after the 12 months Guided Self-Determination program with two patients at risk of COPD, four patients with COPD, three nurses, and five general practitioners. The intervention consisted of structured consultations with the nurse and patient in collaboration with the general practitioner at baseline and after 3, 6, and 12 months. The Guided Self-Determination method comprised facilitation of a mutual reflection process between the patient and the nurse to enhance self-management skills. Each consultation lasted for 60 min. The interviews were analysed using thematic analyses.

**Results:**

Two themes were identified: (1) A structured follow-up is challenging but motivating. (2) A counselling method that opens for conversation, but it requires resources.

**Conclusions:**

The findings indicated that patients with or at risk of COPD experienced enhanced self-management skills after participating in a structured and systematic team-based follow-up in general practice with use of the Guided Self-Determination method. The regularity of the follow-up seemed to be important to succeed to help the patients making lifestyle changes to increase health benefits. However, the Guided Self-Determination method was experienced as time consuming among the general practitioners and nurses, and there are currently no available financial rates for this type of treatment in Norway which may be a barrier to further implementation.

**Trial registration:**

The trial is registered in ClinicalTrials.gov (ID: NCT04076384).

## Key messages regarding feasibility

What uncertainties existed regarding feasibility?

- A main uncertainty regarding this study was if a person-centred Guided Self-Determination intervention among patients with COPD or at risk of COPD was experienced as feasible among patients and healthcare professionals in general practice.

What are the key findings?

- The findings suggest that patients with or at risk of COPD may benefit from a structured and systematic team-based follow-up in general practice. It also indicates that the intervention may facilitate and support the patients’ self-management skills in everyday life.
- The team-based follow-up using this Guided Self-Determination method was seen as time consuming among the general practitioners and nurses, and there are currently no available financial rates for this type of treatment in Norway which may be a barrier of implementation of the treatment in clinical practice.

What are the implications of the findings on feasibility for the design of the main study?

- The Guided Self-Determination method was experienced as useful among both the patients and healthcare professionals in the follow-up of patients with or at risk of COPD in primary healthcare to enhance self-management skills.
- Future studies should include a progression to a RCT pilot study involving a number of general practitioner clinics to examine the potential of Guided Self-Determination as a method to increase self-management in people living with a chronic disease.

## Background

Chronic obstructive pulmonary disease (COPD) is a leading cause of morbidity and mortality worldwide [1, 2]. An upcoming challenge is implementation of cost-effective prevention and management strategies to stem the increasing prevalence of COPD and its costs [3]. Systematic reviews suggests that self-managements interventions among people with COPD might improve health related quality of life and reduce hospital admissions [4–6].

Self-management including smoking cessation, education and coaching should be a major component of the health care delivery system to engage and motivate patients to develop skills to manage their COPD [7]. A systematic literature review suggested that person-centred care may lead to significant benefits for patients in a variety of contexts [8]. However, lack of competence in COPD management among healthcare professionals (HCPs) in primary healthcare seem to be a barrier to the implementation of these strategies [9]. Improving patients’ skills requires tools to enable self-management [10]. Different approaches and models to facilitate a self-management process have been used in previous studies [5, 11, 12]. Motivational interviewing as an approach seems to promote treatment adherence among patients with COPD [11], also interventions using high-resource intensive Cognitive Behavioral Therapy has been used and demonstrated small significant reductions in symptom burden among these patients [13]. Nevertheless, adherence to treatment plans over time is challenging [3] and poor treatment adherence among people with chronic conditions is a problem and the prognosis depends to a great extent on the treatment adherence [14].

Guided Self-Determination (GSD) is a person-centred care approach based on empowerment philosophy, which in turn is based on self-determination theory [15–17]. The GSD is a tool to facilitate problem-solving and mutual decision-making between healthcare professionals and patients [15, 16]. The GSD method has shown effects in life skills and reduction in blood sugar level for patients with type 1 diabetes [17] and also effects in self-management skills among participants comprising endometriosis, haemodialysis, or schizophrenia as well as for people with type 2 diabetes [17–25]. However, the GSD has not been used to increase self-management in people with COPD or at risk of COPD in primary care. Thus, on this background, we designed an interdisciplinary follow-up program in general practice based on the GSD counselling method to facilitate problem-solving and mutual decision-making between healthcare professionals and patients. In the present study, we aimed to explore patients and healthcare professionals experiences with the team-based follow-up program describing areas of uncertainty and important unintended consequences to be addressed. Feasibility issues included the patients` overall experiences in participating, and the nurses and the general practitioners(GPs) experiences of barriers and facilitators of implementation of the GSD intervention. In addition, feasibility issues on how the nurses perceived using the GSD method and the training they had received were addressed.

## Material and methods

### Design

As part of a feasibility study, we used a qualitative study design to explore the participants` and healthcare professionals` experiences after participating in an interdisciplinary GSD follow-up program. The framework for developing complex interventions from the Medical Research Council [26] recommend feasibility studies when testing out a new program such as this interdisciplinary GSD follow-up program. To learn from feasibility studies are important to get enhanced insights to inform larger scale intervention trials [27, 28].

### Setting and participants

Initially, all patients aged 20–80 years who visited one large general practice clinic in Western Norway diagnosed with or identified as at risk of COPD were invited to participate in the interdisciplinary GSD follow-up program. The general practice clinic had to have nurses employed in the staff. The patients visiting the GP clinic during May to June 2019 (*N* = 650) were invited to complete a survey with the aims of identifying patients with or at risk of COPD during a routine scheduled consultation. All patients with COPD (Global Initiative for Chronic Obstructive Lung Disease (GOLD)) stages I–IV [29] and post-bronchodilator forced expiratory volume 1 s (FEV_1_) to forced vital capacity (FVC) ratio < 0.7) were invited by telephone to participate in the 12 months pre-post GSD follow-up program (*N* = 16). In addition, patients who were current smokers and at risk of COPD and motivated to quit smoking were recruited (*N* = 14). The exclusion criteria were severe somatic disease (e.g., severe cancer, severe heart failure, end-stage renal disease), severe psychiatric diagnosis (e.g., severe depression, bipolar disorder, schizophrenia), recorded cognitive deficiency (e.g., Down’s syndrome, Alzheimer’s disorder), or inability to write, speak, or understand Norwegian.

**Fig. 1:**
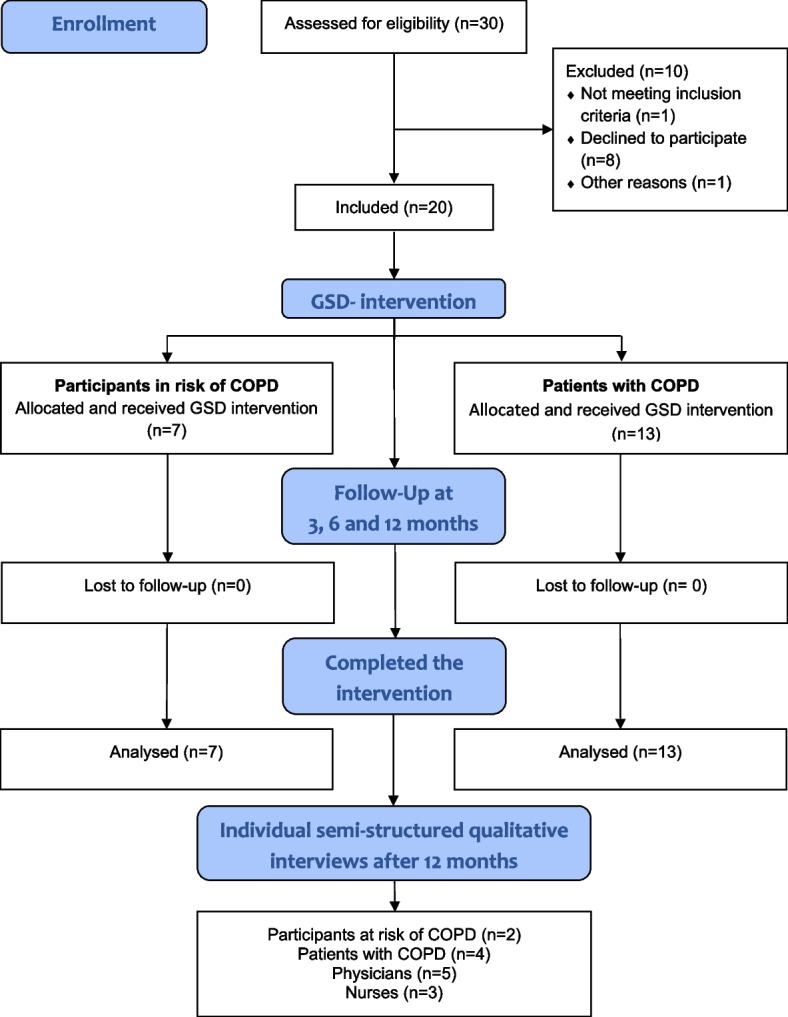
Flow chart of the study. Abbreviations: COPD: chronic obstructive pulmonary disease; GSD: Guided Self-Determination

Twenty patients were included and completed the intervention (Fig. 1). To explore patients and healthcare professionals` experiences with the follow-up program, six patients were invited (five women and one man, mean age 68 years (range 54–80 years)) and all of them participated in individual interviews after the GSD-program. The interviews were conducted during January to March 2021. Two were at risk of COPD, and four had been diagnosed with COPD > 7 years earlier. Three nurses and five general practitioners (GPs) participated in the interviews; their respective work experiences were between 3 and 32 years in general practice.

The study obtained ethical approval from the South-Eastern Norway Regional Committee for Medical and Health Research Ethics (2019/28/REK South-East) and is registered at ClinicalTrials.gov (ID: NCT04076384). The study is reported in accordance with the Consolidated Criteria for Reporting Qualitative Research (COREQ) guidelines [30]. All patients gave informed consent before participation. Further information can be obtained from ClinicalTrials.gov (ID: NCT04076384).

### GSD intervention

The 12-month GSD intervention comprised an interdisciplinary follow-up program based on the GSD counselling method. GSD was used in every consultation between nurses and patients with or at risk of COPD (Table 1). The intervention lasted from November 2019 to January 2021. The patients received structured consultations with a nurse in collaboration with the GP at baseline and after 3, 6, and 12 months. Due to the COVID pandemic, face-to-face consultations were replaced with phone consultations at the 6 months follow-up for some of the patients. Each consultation lasted for 60 min. The focus for the patients at risk of COPD was to quit smoking. These patients received additional 1–2 phone calls during the first month to facilitate smoking cessation. The GPs had overall responsibility for the medical treatment during the study. Depending on each patient’s needs, the GPs attended the consultations after the nurses had undertaken the GSD counselling program, or they were involved when medical decisions or medical assistance were required. After every consultation, written information was documented in the patients’ medical journal, and assessed and approved by the GP.

**Table 1:** Overview of the consultations with use of reflection sheets

Consultations 1–4	Reflection sheets to fill out between the consultations
GSD 1: Your life with the condition	A consultation with focus on: Your life with the disease Invitation to collaborate with the health care professionals
GSD 2: Your life with the condition	A consultation with focus on: At present, what do you find difficult living with the condition? Filling out unfinished sentences about your needs, values, habits and opportunities Can you give a picture, metaphor, or expression of your life with the condition?
GSD 3: Focus for change	A consultation with focus on: Room for the condition in your life Your plans for changing your way of life Clarification of challenge in your life with the condition
GSD 4: Work with changes	A consultation with focus on: Goals and intentions Your thoughts and feelings Your actions New strategies and plans
GSD: Guided Self-Determination	

We modified the original GSD program developed for type 1 diabetes care [15, 16] and adapted worksheets for patients with COPD or at risk of COPD to facilitate reflection and further problem-solving [19–21, 31]. To facilitate for this ongoing empowerment, process the patients should work with specified worksheets between the consultations. These worksheets were discussed and elaborated in the next consultation with the nurse. To secure the quality of the intervention, the nurses participated in a training program to learn advanced GSD communication skills such as mirroring, active listening, and values clarification response. The training program guided the nurses in facilitating a mutual reflection process to enhance self-management skills for patients at risk or with COPD. The nurses training program comprised a reading list to be read before participating in two mini seminars that lasted 3 h each time. In addition, they were participating in supervised patient consultations with a certified GSD nurse a full day.

### Data collection

#### Interviews

After the 12-month GSD intervention, the first author (BCHK) who is a diabetes specialist nurse and experienced in qualitative methods performed semi-structured individual telephone interviews with the participants that lasted for 30–40 min. Telephone interviews were chosen due to the COVID-19 pandemic. In the start of the interviews, the researchers facilitated for a relaxed atmosphere during the interview. Applying semi-structured interviews gave us the opportunity to cover the topics in the study, but it also allowed the participants to express their experiences in their own terms. The semi-structured interview guide was pilot tested on one patient with COPD and one of the nurses not attending the study. The interviews were audio-taped. The patients were asked to share:

- Their overall experience participating in GSD intervention
- Their previous experience with earlier follow-up by the GPs
- How they experienced counselling in the consultations
- Positive and negative feedback

The nurses and the GPs were asked to reflect upon the following:

- Barriers to and facilitators of implementation of GSD
- The training the nurses received and how they perceived using the method

The data collection ended when the researchers did not receive more variation in the interviews and experienced that there was sufficient information to get more insights into the study aim.

#### Demographic and clinical data

Patients’ characteristics were collected at baseline in November 2019 from the patients’ electronic journal including age, comorbidities, and lung function testing with registration of FEV_1_, FVC, and FEV_1_/FVC [32]. The COPD Assessment Test (CAT) [33] was used to address the impact on COPD in daily living. To measure dyspnea the modified Medical Research Council Dyspnea Scale (mMRC) [34] was used.

### Data analysis

The interviews were transcribed and analysed using thematic analysis, as described by Braun and Clarke [35, 36]. (1) The researchers, that had nursing and physiotherapy as professional background (BCHK, MG, CRB, BF) read the data several times to become familiar with the data, coded the data individually, scheduled meetings to discuss the codes further, and thereafter systematized the data by identifying extracts relevant to each code. Using the codes, the topics were reorganised, and new codes were created. Finally, after several discussions, themes were identified and agreed by all authors.

For demographic and clinical data, we used descriptive statistics to compute frequencies and percentages for categorical variables and means and standard deviations (SDs) for continuous variables to examine the impact of the 12-month GSD intervention. The analyses were conducted using IBM SPSS Statistics (version 27).

## Results

### Participants’ characteristics and smoking cessation

**Fig. 2:**
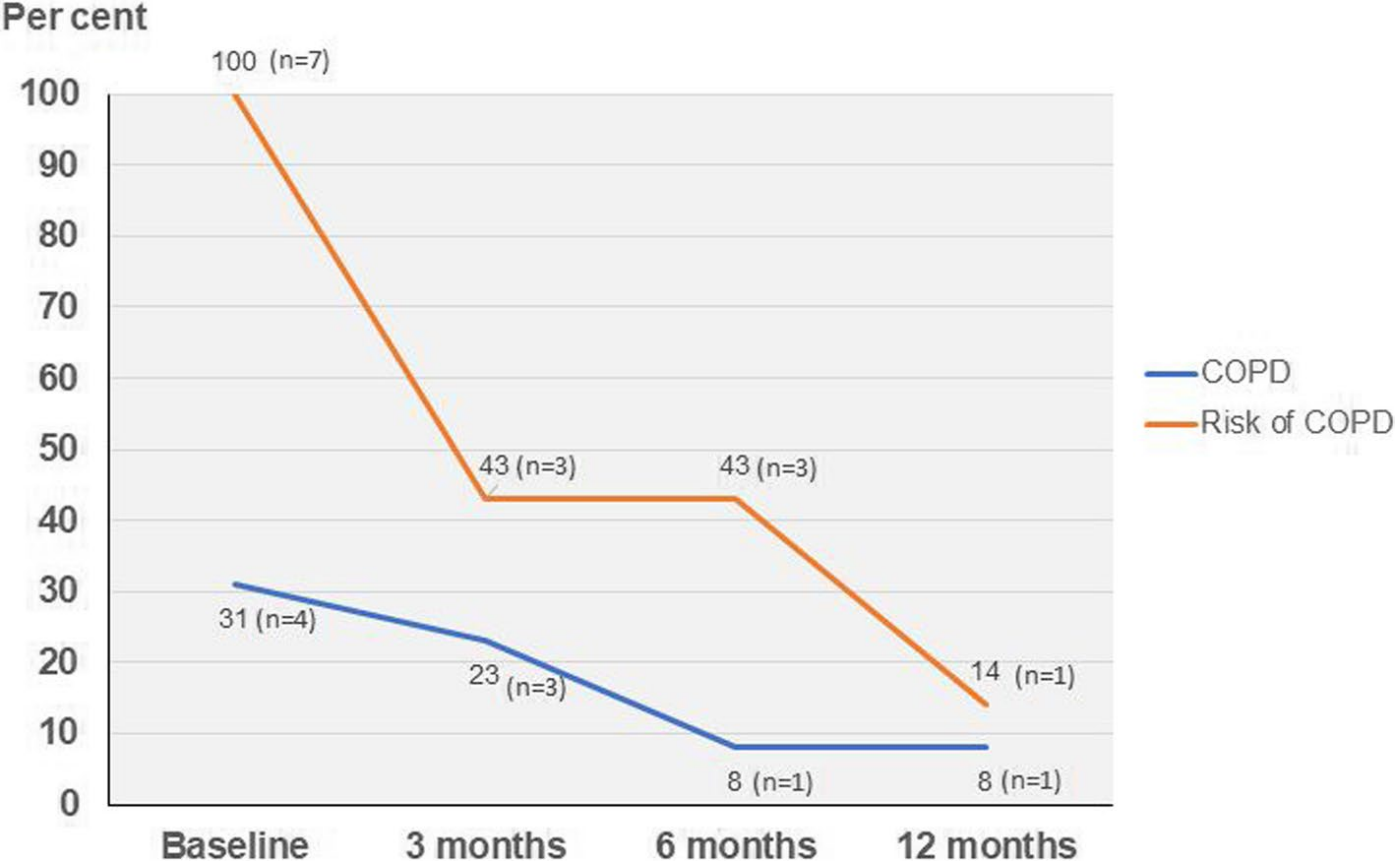
Smoking status for the patients with or at risk of chronic obstructive pulmonary disease (COPD) at the baseline, and at the 3-, 6-, and 12-month follow-ups. Data are expressed as percentage and number of people

Thirteen patients with COPD (mean age 71.7 ± 7.7 years) and 7 at risk of COPD (mean age 54.1 ± 9.9 years) received the 12 months GSD follow-up program (Table 2). The mean total CAT score at baseline in patients with COPD was 10.6 ± 5.6 (Table 2), which indicates a medium impact of the COPD of the overall health and well-being [33]. Of the participants with COPD, nine (69%) were in GOLD stage II or III. Four participants with COPD (31%) were current smokers, and all patients at risk of COPD were smokers at baseline. At 12-month follow-up, one participant (8%) with COPD and one (14%) in the risk group were still smokers (Fig. 2). Thus, after a mutual reflection process between the patient and the nurse to enhance self-management skills, the number of smokers among the patients at risk of COPD and patients with COPD was reduced at 12-month follow-up.

**Table 2:** Characteristics of the patients diagnosed with COPD (*n*=13) and the participants in risk of COPD (*n*=7)

Variables	COPD *n* = 13	Risk of COPD *n* = 7
Sex, female/male (%)	6/7 (46/54)	6/1 (86/14)
Age (years)	71.0 ± 7.7	54.1 ± 9.9
Smoking status *n* (%)		
Never smoked	1 (8)	
Current	4 (31)	7 (100)
Former	8 (61)	
GOLD grade *n* (%)		
I	3 (23)	
II	6 (46)	
III	3 (23)	
IV	1 (8)	
BMI (mean, kg/m^2^)	25.5 ± 3.5	23.3 ± 3.3
FEV_1_ (% pred)	59.1 ± 21.5	74.5 ± 12.2
FVC (% pred)	83.0 ± 19.6	87.5 ± 10.9
FEV_1_/FVC (%)	55.3 ± 11.5	70.0 ± 3.7
CAT total score	10.6 ± 5.6	
mMRC dyspnea grade (median)	1.0	3.38 ± 0.53

Data are presented as mean ± SD, unless otherwise stated. *GOLD* Global Initiative for Chronic Obstructive Lung Disease, *BMI* body mass index; weight/height^2^, *FEV*_*1*_ forced expiratory volume in one second, *FVC* forced vital capacity, *CAT* COPD Assessment test, *mMRC* modified Medical Research Council

### Experiences with the GSD follow-up program

Analysis of the qualitative data produced two main themes: (1) *a structured follow-up is challenging but motivating* and (2) *a counselling method that opens for conversation but* requires *resources*.

#### Theme 1: a structured follow-up is challenging but motivating

The patients felt that the consultations with the nurses were structured and that the time allocated to the GSD was sufficient. It was valued that the nurses encouraged them to be more involved in managing their condition. Some emphasized the usefulness of understanding more about their condition and that the intervention positively affected their life situation. Others welcomed the relationship developed in the consultations between the healthcare professionals` and described it as safe and caring. The intervention made it easier to realize shared decisions about treatment and to balance choices according to patients’ needs, life situation, and medical condition. Some expressed it as follows:

I feel I have increased my knowledge, emotional security and insight into the diagnosis and disease compared to before. It has motivated me to seek knowledge on my own about what it means to have the disease.* (Informant 16)*

Also expressed by some with COPD:

Yes, to set goals—at least to quit smoking—was necessary…… . if no one had said anything you would still have smoked for sure. So, it is probably part of what was said there and how it was done. … motivating yes…. and I knew I was going to come back … so then, you … thought more about it, that you had to quit smoking. (Informant 13)

The conversation with the nurses gave the patients ideas about essential things that could improve their health condition in daily life. This conversation helped to increase the patients’ sense of responsibility for their self-care. One way of saying it was:

When it comes to challenge, it was to try to be active and not be too sedentary. I had to challenge myself a little … Yes, … try a little more, just that, try a little more, try again, yes … I try to push myself a little longer from time to time, a little more, a little more. A bit like … well I can hear the nurse’s voice … so I have that in mind … so it’s something I repeat now that I did not do before. (Informant 7)

The GPs and nurses said that the follow-up of patients differed from what they had experienced before they implemented GSD. The services for patients with or at risk of developing COPD became more systematic and included better management strategies and tools in the consultations. One GP stated:

The bottom line is that the patients get a better service, a better treatment from us. It will not reduce my workload but rather increase it as I must familiarise myself with what happens during the nurses’ consultations. But what we can offer is a systematic and structured treatment for the patients. We now have a systematic follow-up when it comes to lifestyle counselling, and I think that the GPs can learn from the nurses. (GP 1)

The structured follow-up by the nurses was an important contribution to learn from each other’s skills and professional approaches became an essential task.

#### Theme 2: a counselling method that opens for conversation, but it requires resources

The patients perceived that they were seen and heard in the interactions with the nurses. They felt a greater focus on their experience of living with the disease and their life situation. In the patients’ view, both the time and content of the consultations differed from what they had experienced in previous follow-ups. Expressed like this:

I feel much safer, you could say, in relation to the diagnosis or the condition. I dare to ask questions; for example, I have come so far that I asked if it was possible to change my medication. I would never have done that if I was not participating in this study. (Informant 16)

The patients felt they had gained acceptance and recognition of their situation from the healthcare professionals. This was expressed in the following way:

The consultations have been different in terms of what I have been talking about with the nurse in previous consultations, with the GP only. There is a difference, and I think this has been positive. (Informant 12)

As a group, the nurses felt that the GSD was helpful to provide greater continuity in the follow-up of the patients. Nevertheless, they were also concerned of it being time consuming, especially when it comes to training. The nurses noted that the time available for training in the use of the GSD should have been extended to increase their self-confidence in use of the method. The GPs also perceived that the GSD team-based follow-up was challenging in a busy clinical practice. They had concerns that the capacity and logistics was challenging due to the increased workload. In addition, the team-based follow-up did not provide extra payment because of the financial system in Norway does not reimburse nurses for team-based work. One of the GPs said:

Many GPs have a perception that this is expensive and will therefore not participate in a team-based collaboration model because of the financial system. If there were separate reimbursements for these consultations the situation would be different. (GP 3)

However, some of the GPs also drew attention to the positive aspects of working in a team and although it requires more resources, they did not consider the lack of reimbursement as a barrier:

It has not become less work for me, nor more. This model provides a better systematic treatment and opportunities for both the patients and in a societal perspective,… no doubt about that. (GP 1)

## Discussion

The main findings of this feasibility study point out that both patients and healthcare professionals experienced that the GSD follow-up enhanced the patients’ self-management skills in everyday life demonstrated by a reduction from 11 to two smokers among the participants. Further, the GSD team-based follow-up was perceived as positive among the nurses and GPs, but they had some concerns around resources, financial issues, and that the intervention was experienced as time-consuming. The GPs perceived the team-based follow-up as challenging in a busy clinical practice.

Regarding the first theme—*a structured follow-up is challenging but motivating—*the patients experienced that the consultations with the nurses had facilitated involvement and responsibility for everyday living. A person with COPD requires motivational endurance and self-management to cope with setbacks, such as smoking cessation [10]. Motivation is also needed when incorporating physical activity into daily living. The barriers to changes in physical activity and behaviour include the lack of knowledge about physical activity and its benefits and managing feelings of breathlessness and anxiety during exercise [37]. Having systematic counselling tools to use during the follow-up of patients with COPD, especially those relating to helping patients cope with symptoms, are considered by healthcare professionals to be important for helping patients to improve their health [10]. The nurses and GPs in our study noted that the GSD helped them to provide the patients a more structured and systematic follow-up and better management strategies. This is consistent with previous research showing that use of self-management interventions for patients with COPD should include approaches that use behavioural change methods to facilitate patient motivation and activation to develop skills toward enhanced self-management [38]. An advantage of a structured follow-up program is the regular monitoring which seems to provide benefits for patients with COPD [39]. The regularity in the follow-up may help patients maintain lifestyle changes and elicit patient activation, which seems to be key for successful self-management. The new national guidelines in Norway emphasise a structured follow-up in general practice once a year, but more often if symptoms and treatment require a closer follow-up [40].

Although the healthcare professionals were positive about implementing the GSD, they had some concerns around the time and financial issues. Having sufficient time during the consultations with the nurses was one of the reasons noted by patients that stimulated them to become more involved in self-management and to learn more about their illness and how it affects their life and health. Previous research has found that lack of time is a barrier to GPs becoming familiar with COPD guidelines [41] and to providing health promotion to enhance patient self-management [42]. Despite their knowing that the time set aside for the consultation could facilitate self-management, the nurses felt deeply the conflict between the time allotted to patients and the time available in a busy general practice clinic. Knowing that time spent with patients during the follow-up is key to helping them to develop self-management skills may convince practitioners that this is time well spent.

Regarding the theme—*a counselling method that opens conversations*, *but it requires resources,—*the literature notes that a traditional health-care approach, in which the nurses and physicians assume leadership often is taken [43]. Despite the awareness of healthcare professionals about the need to provide patients with support to make lifestyle changes, little attention has been paid to the time constraints when trying to improve patient activation and change of behaviour [44]. Our findings illuminate the importance of considering the patients’ goals and motivation in a shared decision-making approach, such as the GSD. In our study, the patients felt secure and safe, which made it easier for them to become more active in their treatment and lifestyle choices.

However, previous research has shown that simply giving basic information on lifestyle advice is not sufficient to motivate them to start the process of changing lifestyle [9]. Our findings underline the importance of allowing patients to take an active role in the management of their health. For instance, with the GSD approach some patients actively searched for more knowledge. This is consistent with previous research [45] showing that motivated patients are more devoted to change their health behaviour. However, in primary care, it is challenging to provide sufficient follow-up care tailored to each patient. It is previously shown that a team-based approach can facilitate better outcomes for patients with or at risk of developing COPD [46], indicating that a decrease in patient smoking activity during and after counselling sessions is possible. They also reported that a team-based approach with an intensive follow-up contributed to fewer hospital admissions and symptoms after 6 and 12 months among patients with COPD. Our findings support this as the number of smokers decreased in both groups. The interview results showed that smoking cessation was facilitated by the attention to goals, barriers, and motivation in the GSD approach. Thus, a structured and intensive follow-up benefitted the patients.

### Strengths and limitations

Because of the COVID-19 pandemic and the Norwegian lockdown, we were unable to recruit more patients. The GPs did not perform regular follow-up of people with chronic diseases, and face-to-face consultations were replaced with phone consultations at the 6 months follow-up for some of the patients. However, no patients dropped out during the total follow-up of 12 months. Although only six patients participated in the interviews, the heterogeneity according to age and disease severity allowed us to obtain rich and varied data. These insights are important to inform key decisions in the design of future intervention studies implementing the GSD method in general practices.

## Conclusions

The findings indicated that patients with or at risk of COPD, experienced enhanced self-management skills after participating in a structured and systematic team-based follow-up in general practice with use of the Guided Self-Determination method. The regularity of the follow-up seemed to be important to succeed to help the patients making lifestyle changes to improve health benefits. However, the GSD was experienced as time consuming among the GPs and nurses, and there are currently no available financial rates for this type of treatment in Norway which may be a barrier to further implementation. These finding are important to consider when progressing to future intervention studies using GSD as a method in the follow-up of patients in general practice.

## Data Availability

The datasets generated during and/or analyzed during the current study are not publicly available due to ethical and legal restrictions related to confidentiality, the data cannot be deposited online as the study participants have not explicitly been informed about, nor approved data sharing when the data were gathered.
